# Tannic Acid-Dependent Modulation of Selected *Lactobacillus plantarum* Traits Linked to Gastrointestinal Survival

**DOI:** 10.1371/journal.pone.0066473

**Published:** 2013-06-11

**Authors:** Inés Reverón, Héctor Rodríguez, Gema Campos, José Antonio Curiel, Carmen Ascaso, Alfonso V. Carrascosa, Alicia Prieto, Blanca de las Rivas, Rosario Muñoz, Félix López de Felipe

**Affiliations:** 1 Laboratorio de Biotecnología Bacteriana, Instituto de Ciencia y Tecnología de los Alimentos y Nutrición, Consejo Superior de Investigaciones Científicas (ICTAN-CSIC), Madrid, Spain; 2 Dpto. Biología Ambiental, Museo Nacional de Ciencias Naturales, Consejo Superior de Investigaciones Científicas (MNCN-CSIC), Madrid, Spain; 3 Grupo de Microbiología y Biocatálisis de Alimentos, Instituto de Investigación en Ciencias de la Alimentación, Consejo Superior de Investigaciones Científicas (CIAL-CSIC), Madrid, Spain; 4 Dpto. Biología Medioambiental, Centro de Investigaciones Biológicas, Consejo Superior de Investigaciones Científicas (CIB-CSIC), Madrid, Spain; Institut Pasteur Paris, France

## Abstract

**Background:**

Owing to its antimicrobial properties dietary tannins may alter the functional efficacy of probiotic lactobacilli in the gastrointestinal (GI)-tract influencing their growth, viability and molecular adaptation to the intestinal environment.

**Methods and Findings:**

The effects of tannic acid on *Lactobacillus plantarum* WCFS1 were studied by *in vitro* growth monitoring and visualizing the morphological alteration on the cell wall using transmission electron microscopy. Growth upon tannic acid was characterized by dose-dependent reductions of initial viable counts and extended lag phases. Lag phase-cells growing upon 0.5 mM tannic acid were abnormally shaped and experienced disturbance on the cell wall such as roughness, occasional leakage and release of cell debris, but resumed growth later at tannic acid concentrations high as 2.5 mM. To gain insight on how the response to tannic acid influenced the molecular adaptation of *L. plantarum* to the GI-tract conditions, gene expression of selected biomarkers for GI-survival was assessed by RT-qPCR on cDNA templates synthetized from mRNA samples obtained from cells treated with 0.5 or 2 mM tannic acid. Tannic acid-dependent gene induction was confirmed for selected genes highly expressed in the gut or with confirmed roles in GI-survival. No differential expression was observed for the *pbp2A* gene, a biomarker negatively related with GI-survival. However PBP2A was not labeled by Bocillin FL, a fluorescent dye-labeled penicillin V derivative, in the presence of tannic acid which suggests for enhanced GI-survival reportedly associated with the inactivation of this function.

**Conclusions:**

Probiotic *L. plantarum* WCFS1 is able to overcome the toxic effects of tannic acid. This dietary constituent modulates molecular traits linked to the adaptation to intestinal environment in ways previously shown to enhance GI-survival.

## Introduction

Western populations are currently facing the increased incidence of gut dysbiosis (an imbalance in the intestinal bacteria leading to disease) and a loss of gut microbial richness [1] outcome, among other life-style variables, of the consumption of typical western diets high in animal fat, sugars and calorie-dense foods [2]. Given that host diet plays a determinant role to maintain and define the composition of gut microbiota, dietary habits modification may be a powerful tool to induce changes in the gut microbial composition to benefit host health.

The promotion of fruit, vegetable and fibre consumption in western populations intends to change dietary habits to prevent gastrointestinal cancer and cardiovascular diseases [3]. Fruit, legumes and leafy vegetables are main sources of tannins which are polyphenols believed to be involved in chronic disease prevention [4]. Owing to their antimicrobial properties [5] tannins may induce transformation changes in our gut microbiota. Supporting this view, a previous report have shown evidence that red wine polyphenols increase *Lactobacillus* and *Bifidobacterium* populations in the colon content of rats [6], two bacterial groups inherently resistant to tannins [7] and considered beneficial for the intestinal function [8].

In order to leverage information regarding how dietary tannins influence the gut microbiota, a deeper knowledge is required about the effects produced by these polyphenols on gut microorganisms and the mechanisms used to withstand the inhibitory effects of these micronutrients. This information is important to select tannin-resistant gut probiotic microorganisms that potentially improve our adaptation capacity to customized diets and contribute for a better management of our gut ecosystem and host health. A recent study have deciphered tannin resistance mechanisms in *Lactobacillus plantarum* [9], the sole tannin degrading bacteria of human origin found in a previous search for tannin-degrading bacteria from human faeces [10]. Tannins diminished the synthesis of proteins involved in the cyclopropanation of membrane lipids, stress response at population scale and maintenance of cell shape while increased the synthesis of proteins involved in oxidative stress defence and cell wall biogenesis [9]. In addition it was reported that tannic acid-adapted cells of *L. plantarum* submitted to a medium containing tannic acid displayed a prolonged viability during stationary phase [11]. The upregulated proteins were mainly related to the energy metabolism (glycolysis) and protein-synthesizing capacity (ribosomal proteins).

Given that functional efficacy in the GI-tract of a particular microorganism depends in part on its numerical abundance and viability, in this work we investigated how tannic acid influences the growth and morphology of a gastrointestinal isolate of *Lactobacillus plantarum*. Likewise, the marked impact of host diet on the different responses displayed by *L. plantarum* to the gut environment [12] led us to ask whether tannic acid contributed to modulate selected molecular traits reportedly involved in the adaptive response of *L. plantarum* to the gut environment. The examined traits included recently discovered markers of *L. plantarum* gastrointestinal robustness [13], exopolysaccharide (EPS) production and a selection of genes observed to be induced during intestinal passage in previous human [14], [15] or mice [16], [17], [18] studies.

## Materials and Methods

### Bacterial strain and culture conditions

*Lactobacillus plantarum* WCFS1, a probiotic strain isolated from human saliva [19] was cultivated in MRS [20] or modified semi-synthetic RPM media [21] at 30°C. Tannic acid (T0125) was obtained from Sigma (Madrid. Spain). A 100 mM tannic acid stock solution was prepared in 5% (w/v) acetone. To study the effects of tannic acid on growth, RPM was used as medium of choice since MRS proteins precipitated in the presence of 1 mM tannic acid (not shown). Overnight MRS cultures were used to inoculate fresh RPM medium containing tannic acid at 0.125, 0.25, 0.5, 1 or 2.5 mM (final concentration). To rule out an effect of the acetone used for tannic acid preparation, all control cultures running in parallel with the tannic acid-amended cultures contained equivalent amounts of acetone.

These cultures, inoculated RPM devoid of tannic acid and their respective cell free controls, were incubated at 30 °C under static conditions. Bacterial growth was quantified on MRS agar plates by serial dilution and viable colony counts from periodically collected samples. Growth experiments were performed in triplicate. At least 2 replica plates at each time point were used to construct the growth curves.

### Transmission electron microscopy

Growing cells were recovered in the lag phase (16 h for 0.5 mM tannic acid-amended RPM cultures; 4 h for control cultures lacking tannic acid). For transmission electron microscopy (TEM) analyses, these bacteria were centrifuged at 7,500 x g for 10 min. The cells were suspended in a fixative solution containing 3.25% glutaraldehyde in phosphate buffer (100 mM, pH 7.1). In the experiments aimed to detect polysaccharides over the outer surface of cells, 0.15% (w/v) ruthenium red was also added to the fixative solution. The cell suspensions were incubated for 3 h at 4°C and centrifuged at 7,500 x g for 10 min, washed three times in phosphate buffer (100 mM, pH 7.1) and placed in 2% fluid agar (w/v) and immediately homogenized. The solidified agar was cut into small pieces and washed 16 h in phosphate buffer (100 mM, pH 7.1). The pieces containing cells were then fixed for 5 h with 1% osmium tetroxide in 50 mM sodium phosphate buffer and subsequently dehydrated through a set of graded ethanol solutions. After the dehydrating in ethanol, the pieces were placed in propylene oxide, followed by propylene oxide-Spurr resin, and finally were embedded in pure Spurr resin [22] and polymerized at 70°C for 24 h. Ultrafine sections (70–90 nm) of the preparation obtained with a diamond knife in an ultramicrotome Reichert Ultracut E, were placed in copper grids and stained with 1% lead citrate [23] to be examined using a Zeiss EM910 transmission electron microscope at an accelerating voltage of 80 kV.

### RNA isolation, RT-PCR and Real Time qPCR

For RNA isolation, 50 mL MRS cultures of *L. plantarum* WCFS1 were grown up to an OD_600_ of ≈ 1 and then supplemented with tannic acid at final concentrations of 0.5 or 2 mM. After 10 min incubation the cultures were immediately processed for RNA extraction as previously described [24]. Two treatments with DNase I (Ambion) were applied to RNA samples. The RNA quality was verified by PCR by using primers (5′-CAGGCCTAACACATGCAAGTC) and (5′-GGGCGGWGTGTACAAGGC) encoding 16S rRNA gene. The lack of amplified products confirmed the absence of genomic DNA in the RNA preparation. PCRs including or not *L. plantarum* WCFS1 DNA template were carried out and used as positive and negative controls, respectively.

The DNA-free RNA was retrotranscribed using the High Capacity cDNA Reverse Transcription Kit (Applied Biosystems, 4368814) according to the manufactureŕs instructions. From the cDNA obtained, quantitative gene expression was analyzed in an Abi Prism 7500 Fast Real Time PCR system (Applied Biosystems, Foster City, Calif.). Specific primers pairs were designed with the *Primer Express* 3.0 program to amplify internal regions of target genes (Table S1). The expression levels of eight candidate endogenous control genes were evaluated using NormFinder analysis and the most stable, *dnaG*, was used as endogenous control. Amplifications were performed in triplicate as previously described [25]. During RT-qPCR, controls were included to verify the lack of DNA or RNA contamination in the reagents. All real-time PCR assays amplified a single product as determined by melting curve analysis and by electrophoresis. A standard curve was plotted with cycle threshold (Ct) values obtained from amplification of known quantities of cDNAs and used to determine the efficiency (E) as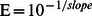. In order to measure *L. plantarum* gene expression, amplification of the endogenous control gene was performed simultaneously and its relative expression compared with that of the target gene. Results were analysed using the comparative Ct method (also named double delta-delta Ct (2 ^ΔΔCt^) method). Genes were considered differentially expressed when a fold change (FC) equal or higher than ±1.5 was observed compared to control.

### Isolation and characterization of the extracellular associated matrix

For extracellular polysaccharide isolation *L. plantarum* WCFS1 RPM cultures of 1L were grown in the presence or absence of 0.5 mM tannic acid for 72 and 24 h, respectively. After centrifugation at 7,500 x g for 25 min the cells were discarded and polyvinylpyrrolidone (PVPP) (1% w/v) was added to the supernatant to remove the tannic acid remaining in the medium. The mix was incubated for 30 min and then filtered through a filter paper. Two volumes of cold ethanol (4° C) were added to the filtered supernatant and the mix incubated 16 h at 4°C for polysaccharide precipitation. After incubation the mix was centrifuged (7,500 x g for 25 min), dried at room temperature and suspended in 30 mL of sterile water. The polysaccharide was dialyzed overnight against distilled water by using a dialysis membrane (3,500 Da pore size) and the pellet lyophilized and kept for further analyses.

### Determination of monosaccharide composition

The polysaccharide samples were hydrolyzed with trifluoroacetic acid for 1 h at 120°C. The monosaccharides obtained from hydrolysis were converted into their corresponding alditol acetates [26] and then identified and quantified by gas-liquid chromatography in an Autosystem (Perkin-Elmer, USA) using an SP-2380 fused silica column (30 m×0.25 mm I.D. x 0.2 µm film thickness) with a temperature program (210 to 240°C, initial time 3 min, ramp rate 15°C/min, final time 7 min) and a flame ionization detector.

### Penicillin binding protein (PBP) detection

RPM cultures containing either 0.5 mM tannic acid or lacking this polyphenol (controls) were inoculated with *L. plantarum* WCFS1 cells and grown to late exponential phase (24 and 60 h for control and tannic acid-amended cultures, respectively). The cells were harvested by centrifugation at 7,500 x g for 15 min and suspended in 3 mL of potassium phosphate buffer (50 mM, pH 6.5). The cells were disrupted by a double passage through a French Press (Amicon French Pressure Cell, SLM instruments) set at 1100 psi. The suspension was then incubated in the presence of 25 µM fluorescent penicillin Bocillin FL (Invitrogen) at 37°C for 15 min. The proteins in the sample were separated by SDS-PAGE on gels containing 10% polyacrylamide. The labeled PBPs were detected on the gel by fluorography in a Fluorescent Image Analyzer (Fujifilm FLA-3000; excitation 488 nm, emission 533 nm).

## Results

### Concentration dependent effects of tannic acid

**Figure 1:**
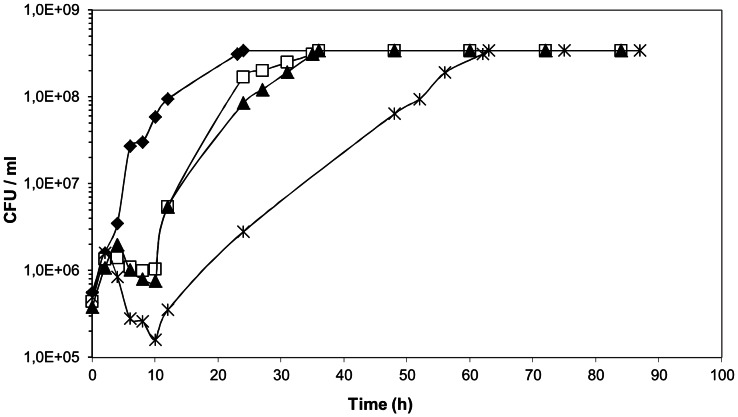
Effects of tannic acid on the growth of *L.* *plantarum* WCFS1. Cells were grown in RPM media supplemented with increasing tannic acid concentrations. Control, ⧫; 0.1 mM tannic acid, □; 0.25 mM tannic acid, ▴; 0.5 mM tannic acid, X. CFU, colony-forming units. Representative curves are shown. Values at each time point are the average of at least 2 replica plates. Standard deviations (not shown) were less than 10%.

Examination of representative growth curves of *L. plantarum* WCFS1 (Fig. 1) revealed that the length of lag phase increased progressively with increasing tannic acid concentration in the medium. In addition, it was observed that a gradual increase of tannic acid resulted in a stepwise decrease of cell counting in the lag phase of growth (about 1 log reduction at 0.5 mM). However, even if tannic acid killed part of the *L. plantarum* population after inoculation, a cell subpopulation adapted to tannic acid challenge and resumed growth at tannic acid concentrations high as 2.5 mM (not shown).

### TEM observation of morphological changes produced by tannic acid

**Figure 2:**
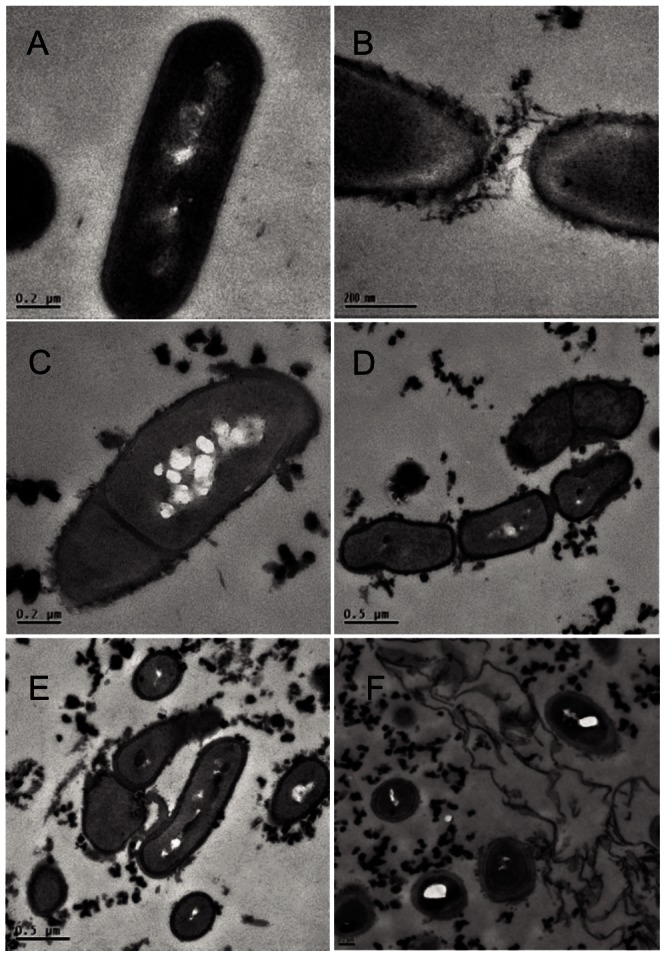
Transmission electron micrographs of lag phase-cells of *L.* *plantarum* WCFS1 treated with tannic acid. Cells were grown for: 4 h in RPM medium lacking tannic acid (A); 16 h (B, C, D, E, F) in RPM medium containing 0.5 mM tannic acid. Scale bar 0.2 µm (A, B, C, F); 0.5 µm (D, E).

To get more insight into the effects of tannic acid on the *L. plantarum* physiology, the cell morphology of lag phase-recovered cells was investigated by TEM. Transmission electron micrographs of untreated WCFS1 cells showed the characteristic rod-shaped morphology with smooth surface and well defined membrane and cell wall (Fig. 2A). When cells from tannic acid-amended cultures (0.5 mM) were visualized, the membrane and cell wall were less defined than in the non-treated cells, cell surfaces appeared less smooth (Fig. 2B) and displayed roughness probably due to leakages. Furthermore, cells that were abnormally shaped and sized (Fig. 2D) or that displayed asymmetric (Fig. 2C) or incomplete (Fig. 2D) divisions, could be observed. Occasionally, a partial collapse of the cell wall was observed and more debris was released from the cell (Fig. 2E).

### Analysis of extracellular matrix production

Visualization by TEM of lag phase-cells growing upon tannic acid occasionally showed the presence of extracellular matrix (Fig. 2F) which displayed a negative ruthenium red staining in the electron microscopy analyses (data not shown). However the observed material was isolated and further analysed since extracellular polysaccharides (EPSs) have been shown to be involved in shielding cell envelope embedded host receptor ligands of the WCFS1 strain [27] or to form a protective shield in *Lactobacillus rhamnosus* GG against host defence molecules [28].

The amount of precipitated extracellular material (0.020 g/L) from WCFS1 cells grown on tannic acid was slightly lower compared to the material obtained from cells grown in the absence of this polyphenol (0.024 g/L). The analysis by GC and GC-MS of alditol acetate derivatives of the carbohydrates precipitated showed that the sugar content in this material was less than 22% and that the sugar primarily present was glucose, regardless of the presence of tannic acid in the media. These results do not support, in the conditions applied in this study, the biosynthesis of an extracellular polysaccharide that accumulated over the outer surface of *L. plantarum* in response to tannic acid. Regarding the observed extracellular layer, which was unrelated to the presence of tannic acid, it could correspond to a dispersed slime which is often secreted by microorganisms consisting of a loose network of unordered fibrils that can be sloughed off into the aqueous phase [29].

### Tannic acid-dependent expression analysis of selected *L. plantarum* genes associated to GI-tract survival

The tannic acid-induced injury on the cell surface shown above fits the increased synthesis of proteins involved in cell wall biogenesis reportedly observed upon tannic acid stress [9]. Given the modified expression of genes coding for cell wall precursors and cell wall proteins is a common functional response to exposure to bile [30] or intestinal passage [14], [16], we wondered if the response of *L. plantarum* to tannic acid also modulated genes coding for cell surface traits expressed in the GI-tract or involved in the adaptive response of this microorganism to the gut environment. To this goal, a selection of genes was made of which expression was followed by RT-qPCR. Selected genes were observed to be induced during intestinal passage in previous human [14], [15] or mice [16], [17], [18] studies and also included recently validated genes that correlated with gastrointestinal robustness of *L. plantarum* [13]. The studied genes and their regulation following a 10 min challenge with 0.5 or 2 mM tannic acid are depicted in Table 1.

**Table 1:** Expression of selected *L. plantarum* WCFS1 genes upon tannic acid challenge.

Group	Locus Taga	Function	Fold changeb,c in cells treated with:	
			0.5 mM tannic acid	2 mM tannic acid
Genes linked to GI-tract survival	*lp_0775* (*argG*)	argininosuccinate synthase	**1.93**	**2.76** e
	*lp_3055 (copA*)	copper transporting ATPase	**1.71**	**2.86** e
	*lp_3473* (*ram2*)	alpha-L-rhamnosidase	**2.34**	**3.19** e
	*lp_2940*	cell surface protein, LPXTG-motif cell wall anchor	**2.05**	**4.39** e
	*lp_1669* (a*raC*)	AraC family transcriptional regulator	0.82	n.d.
	*lp_1413* (*pbp2A*)d	transpeptidase-trans-glycosylase (penicillin binding protein 2A)	0.86	0.87
	*lp_2827* (*napA3*)	Na(+)/H(+) antiporter	**2.17** f	n.d.
PBP-encoding genes	*lp_1413* (*pbp2A*)d	transpeptidase-trans-glycosylase (penicillin binding protein 2A)	0.86	0.87
	*lp_1568* (*pbp2B1*)	transpeptidase (penicillin binding protein 2B1)	1.17	n.d.
	*lp_175 1 (pbp1A)*	transpeptidase-trans-glycosylase (penicillin binding protein 1A)	0.65	n.d.
	*lp_1619 (pkn1)*	serine/threonine protein kinase	0.51	n.d.
	*lp_2200 (pbp2B2)*	Transpeptidase (penicillin binding protein 2B2)	1.39	n.d.
Other	*lp_2790 (serA)*	2-hydroxyacid dehydrogenase	**1.75** g	n.d.
	*lp_0203 (serA)*	C-terminal ACT (regulatory)	**1.98** e	n.d.
	*lp_2956* (*tanLp1*)	tannin acylhydrolase	**4.31**	**12.00** e

a Designated gene number for the annotated *L. plantarum* WCFS1 genome.

b Mean of fold change (FC) of genes selected in *L. plantarum* cultures grown in MRS supplemented with tannic acid (test) relative to growth in MRS without supplement (control). Data are for three independent cultures. n.d., not determined.

c Genes were considered differentially expressed (highlighted in boldface type) when nominal *p-values* were <0.1 and had a fold change (FC) equal or higher than ±1.50.

d *pbp2A* is doubly grouped because is a putative PBP-encoding gene linked to GI-survival (see text and [13]).

e The relative expression level of the target gene is significantly different from the level observed in MRS lacking tannic acid at a level p<0.1.

f Significantly different at a level p<0.05.

g Significantly different at at a level p<0.005.

The expression of *copA* and *lp_2940* (cell surface protein), two genes previously identified to be highly induced in the GI-tract of mice [18] or humans [15] and confirmed to play an important role to the persistence and survival of *L. plantarum* WCFS1 in mice [17] were significantly up-regulated upon 0.5 mM tannic acid addition compared to controls. A significant induction by tannic acid was also observed for *argG* and *ram2,* both genes highly induced in the GI-tract [15], [18], under high osmolarity conditions (*ram2*) [16] or exposed to bile (*argG*) [30]. The impact of tannic acid on the expression of three genes recently validated as markers of *L. plantarum* gastrointestinal robustness [13], which display a negative correlation with enhanced survival in the intestine, was also examined. Two of these genes, i.e. *lp_1669* coding for an AraC-family transcription regulator and *lp_1413* coding for the penicillin-binding protein PBP2A showed no significant modulation, while *lp_2827,* coding for a Na^+^/H^+^ antiporter, was significantly induced upon tannic acid treatment.

Because changes in cell envelope occur as adaptation to the harsh gut environment [15], [16], the expression of *serA* gene (*lp_2790*) upon tannic acid exposure was examined. This gene is highly induced in the human intestine [15] and involved in the biosynthesis of serine, an abundant amino acid in the *L. plantarum* cell-envelope proteins. We observed that *lp_2790* and *lp_0203* (another *serA* paralog overexpressed upon *p*-coumaric acid stress [31]) were significantly induced by tannic acid. We also examined the expression of the tannin acylhydrolase (tannase) encoded by *tanLp1* (*lp_2956*) [32] as it is a molecular trait that might enhance the GI-survival of *L. plantarum* on tannin-containing diets. As shown in Table 1 *tanLp1* expression was notably induced in a dose-dependent fashion by tannic acid.

To further corroborate the effect of tannic acid on the expression of genes playing an important role during the residence of *L. plantarum* in the intestine, we performed a second round RT-qPCR to quantify *argG, copA, ram2, lp_2940* and *lp_1413* transcripts from cells challenged with a higher tannic acid concentration (2 mM). As shown in Table 1 these genes were further up-regulated, especially *lp_2940*, compared to the control. The only exception was *lp_1413* (*pbp2A*) which was not significantly modulated by either of the tannic acid concentrations examined compared to their controls.

### Tannic acid modifies the Penicillin Binding Protein (PBP) profile of *L. plantarum*

As mentioned above *pbp2A* showed no modulation by tannic acid, however we further investigated this function in view that *pbp2A* deletion derivatives of *L. plantarum* displayed the highest relative GI-tract survival among several genes linked to this trait [13]. The penicillin binding proteins (PBPs) can be targeted by antibiotics, such as those belonging to the β-lactam class, as these proteins play a key role in the peptidoglycan biosynthesis and thus are essential for the survival and growth of the bacteria [33]. Antibiotic resistant bacteria can circumvent these inhibitory effects by modifying their PBP profile. To test if tannic acid influenced the PBP profile of *L. plantarum* Bocillin FL, a fluorescent dye-labelled penicillin V derivative, was used.

**Figure 3:**
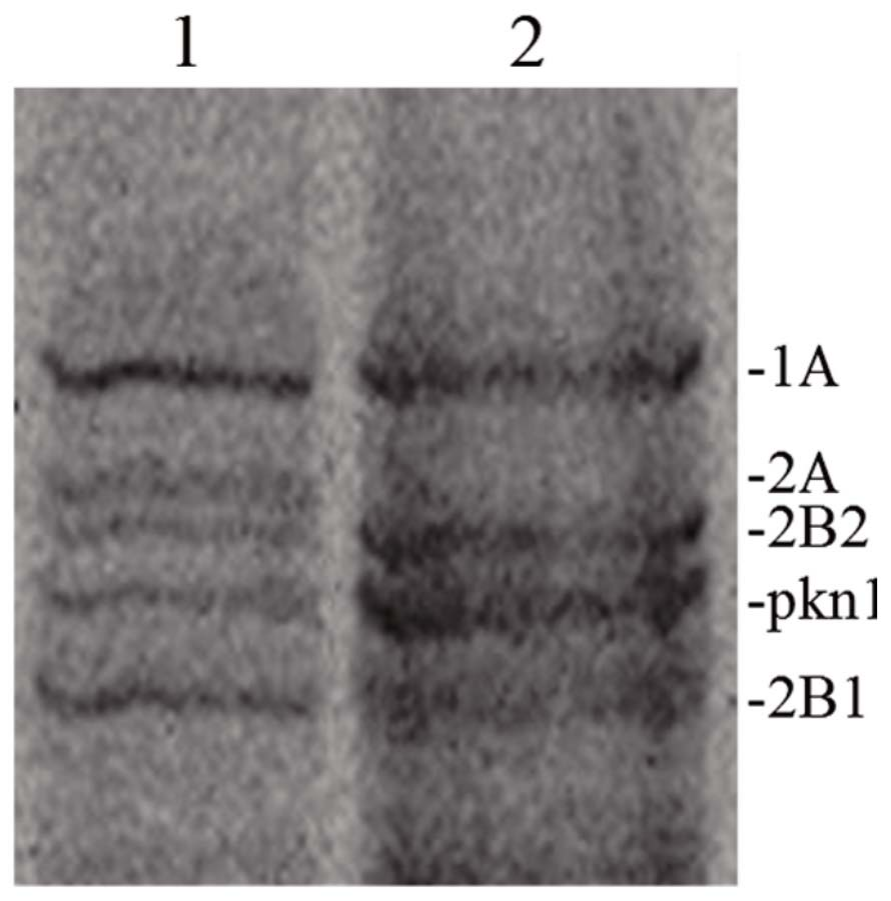
Effect of tannic acid on PBPs from *L.* *plantarum* WCFS1 PBPs were extracted from *L. plantarum* WCFS1 cells grown in absence (1) or in presence (2) of 0.5 mM tannic acid and labelled with Bocillin FL. These proteins were separated by SDS-PAGE and detected on the gel by fluorography (see Material and methods). Based on their molecular size PBPs were named according to the PBPs annotated in the complete genome sequence of *L. plantarum* WCFS1 (see text).

Examination of the bocillin-labelled PBPs of *L. plantarum*, separated according to their molecular size by SDS-PAGE (Fig. 3), revealed that cells grown in the absence of tannic acid synthetized five PBPs with molecular masses matching with those of the PBPs annotated in the complete genome sequence of *L. plantarum* WCFS1, i.e., PBP1A (83.1 kDa), PBP2A (77.7 kDa), PBP2B2 (77.2 kDa), pkn1 (74.5 kDa) and PBP2B1 (72.6 kDa). However, growth of *L. plantarum* WCFS1 cells in media containing tannic acid resulted in the lack of detection of a protein with a mass of approximately 77.7 kDa (PBP2A) thus rendering a PBP profile that displayed only four of the five PBPs originally detected.

In order to distinguish between reduced PBP expression and PBP inhibition, the influence of tannic acid on the expression of the genes encoding for PBP1A (*lp_1751*), PBP2A (*lp_1413*), PBP2B2 (*lp_2200*), pkn1 (*lp_1619*) and PBP2B1 (*lp_1568*) was examined by RT-qPCR. The results showed that tannic acid does not modulate the gene expression of the *L. plantarum* PBPs (Table 1). Notably, *pbp2A* expression was not altered at two different tannic acid concentrations revealing that the absence of bocillin-labelling of PBP2A was not due to reduced PBP2A production.

## Discussion

Dietary regimes are known to play determinant roles over other possible variables to define the human gut microbial composition [2] and have been shown to deeply affect the way a gastrointestinal isolate of *L. plantarum* responds to the mouse gut environment [12].

This study shows how tannic acid, a dietary constituent with antimicrobial properties, affected some physiological characteristics of a *L. plantarum* strain isolated from the GI-tract that are relevant for its adaptation to the gut environment. Since the functional efficacy in the GI-tract of a particular microorganism depends in part on its numerical abundance, the effect of tannic acid on the viability of this model strain was assessed. The observed lengthening of lag phase in WCFS1 cells induced by tannic acid is consistent with the results noted by others for non-commensal *L. plantarum* strains [11], [21] or *Lactobacillus hilgardii* [34]. In this work a tannic acid-dependent decrease of viability was observed which coincided with severe changes in morphology and injuries in the cell envelope of lag phase cells. The cell surfaces of damaged cells were rougher than non-treated cells leaving severe leakages that lead to cellular content losses and probably disturbed the energy balance. In spite of these disturbances on the cell wall, seemingly a subpopulation of *L. plantarum* WCFS1 survived the tannic acid challenge. Beside the increased synthesis of proteins involved in oxidative stress defense [9], the ability to regain growth after the injury induced by tannic acid could be attributable to an increased synthesis of some key proteins involved in cell wall biogenesis observed previously for this microorganism upon tannic acid stress [9]. This functional response parallels with the transcriptional responses displayed by *Escherichia coli* to tannins, which aimed to maintain the integrity of the cell membrane [35], [36]. In addition, the tannic acid-induced leakage of cellular materials to the extracellular milieu observed by TEM might be a contributing factor to restore cell growth as *L. plantarum* produces intracellular tannase to hydrolyze tannic acid [32], even more judging by the clear tannic acid-dependent induction of *tanLp1* observed in this work.

In view of the negative effects of tannic acid on the viability and cell wall integrity of lag phase WCFS1 cells, it is intriguing that murine models fed with red wine polyphenols containing tannic acid with various degrees of polymerization markedly increased the population of lactobacilli in the colon content [6]. Supporting this apparently positive inter-relationship between dietary tannins and survival of *Lactobacillus ssp.* to the gut conditions it was reported that specific strains of *L. plantarum* found in grape must, predominate in the intestinal tract of vinegar flies collected from a winery [37]. This apparent inconsistency, and the fact that the impact of dietary tannins on the molecular mechanisms modulating the adaptive responses of *L. plantarum* to the gut environment remains obscure, aimed us to investigate the influence of tannic acid in the expression of previously reported GI-tract survival traits.

The EPSs have been shown to improve the GI survival of *L. plantarum* WCFS1 [27] and other *Lactobacillus* spp. such as *L. rhamnosus* GG [28], or to act as a protective barrier for some tannin-resistant ruminal streptococci such as *Streptococcus gallolyticus* against the antimicrobial action of tannic acid [38]. However these protective functions provided by EPS have not been yet demonstrated for many *Lactobacillus* sp. while a deliberate increase of EPS synthesis in *Streptococcus bovis* (other ruminal bacterium) did not enhance tolerance to tannic acid [38] highlighting species differences in survival mechanisms. Under the conditions applied in this study we observed that extracellular polysaccharide was not accumulated over the outer surface of *L. plantarum* in response to tannic acid. This result agrees with the decreased synthesis of proteins involved in biofilm formation in this bacterium when submitted to tannic acid stress [9]. In support of this observation galloylated catechins, which are structurally related to tannic acid, also reduced slime production and inhibited biofilm formation in staphylococcal cell cultures [39].

Not only the injuries and morphological changes provoked by tannic acid on the cell wall resembled those induced by bile [30] in *L. plantarum* but also the modified expression of genes coding for peptidoglycan precursors and cell membrane is a common functional response to exposure of bile [30], intestinal passage [14], [18] and tannic acid [9]. Given these parallels we examined the influence of tannic acid on the expression of selected genes coding for cell envelope functions expressed in the GI-tract or involved in the adaptive response of this microorganism to the gut environment.

Tannic acid increased the expression of a*rgG, copA, ram2 and lp_2940*, four genes markedly induced in the GI-tracts of human [15] and mouse [18] where *copA* [15] and *lp_2940* [17] provide confirmed key roles to the persistence and survival of *L. plantarum* in the mouse digestive tract. Notably we were able to observe a tannic acid-dependent up-regulation of these genes among which *lp_2940* was the most induced. The induction of these genes suggests that the response of *L. plantarum* to tannic acid improves survival of *L. plantarum* in the GI-tract. Up-regulation by tannic acid of two *serA* paralogs which code for phosphoglycerate dehydrogenase (a key function for the biosynthesis of serine, a highly abundant amino acid residue in cell-envelope proteins from *L. plantarum*) suggests for an increase of the biosynthesis of cell-envelope proteins which could provide protection on the gut environment.

In addition to these responses, tannic acid differently affected the expression of three specific *L. plantarum* genes with confirmed importance for GI-tract survival [13]. The lack of modulation of *lp_1669*, which was associated to capsular polysaccharide remodeling [13], fits well with the absence of polysaccharide biosynthesis upon tannic acid exposure observed here, suggesting that tannic acid does not influence GI-survival via EPS production or CPS remodeling. According to its proven role in salt tolerance [13] the transcription of *napA3* encoding for a Na^+^/H^+^ antiporter could have been increased to counteract potential osmotic upshifts triggered by the injuries caused by tannic acid on the cell envelope. However, under stomach acidic conditions the activity of NapA3 has been suggested to increase proton influx to decrease internal pH and proton motive force (pmf) which explain the improved GI-tract survival of *napA3* deletion derivatives [13]. Thus we suggest that NapA3 is probably involved in osmotolerance *in vivo* unless the pH is too low so that the proton influx becomes a negative factor to maintain pmf and pH homeostasis. The *pbp* gene expression, including *pbp2A*, was not altered at two different tannic acid concentrations but PBP2A was the sole PBP not labelled by bocillin suggesting that tannic acid inactivated the protein. Regardless the effects on *lp_1669* and *napA3*, PBP2A inactivation by tannic acid would contribute to improve the relative GI-tract survival of *L. plantarum* since the positive effects of these deletion derivatives on GI robutness are not cumulative [13].

In conclusion, this study shows the capacity of a *L. plantarum* strain isolated from the GI-tract to overcome the toxic effects of tannic acid. The response of *L. plantarum* to this dietary compound includes the modulation of selected biomarkers involved in the molecular adaptation to the intestinal environment in ways previously shown to enhance GI-survival. These results improve our understanding of the contribution of dietary compounds on gut microoganisms-host interactions. This knowledge may be promising towards the selection of probiotic tannin-resistant microorganisms with enhanced GI-survival that potentially improve our adaptation capacity to customized diets and contribute for a better management of our gut ecosystem and host health.

## Supporting Information

Table S1 **Oligonucleotides used for RT-qPCR in this study designed with the Primer Express 3.0 software.** ^a^ Designated gene number for the annotated *L. plantarum* WCFS1 genome. ^b^ (5′→ 3′). ^c^ Internal control gene used to calculate the relative expression.
(DOC)
