# Supplementary material for: Tannic Acid-Dependent Modulation of Selected Lactobacillus plantarum Traits Linked to Gastrointestinal Survival
Source: PLoS One. 2013 Jun 11;8(6):e66473. doi: 10.1371/journal.pone.0066473 (PMC3679024; doi:10.1371/journal.pone.0066473)
Supplement: Table S1 — Oligonucleotides used for RT-qPCR in this study designed with the Primer Express 3.0 software. a Designated gene number for the annotated L. plantarum WCFS1 genome. b (5′→ 3′). c Internal control gene used to calculate the relative expression. (DOC) [file pone.0066473.s001.doc]

| **Locus Tag^a^** | **Description** | **Forward primer sequence^b^** | **Reverse primer sequence^b^** | **References** |  |
| --- | --- | --- | --- | --- | --- |
| *lp_0203* | C-terminal ACT (regulatory) | AACACCCCGGGAAGTAAC | AATGAGCAGGCCGATGATG | 8 |  |
| *lp_0775* | argininosuccinate synthase | AAAGCTTGCGGATACGATCTACA | CCATCGCTTCCATCAATGG | 14, 17 |  |
| *lp_1413* | transpeptidase-trans-glycosylase (penicillin binding protein 2A) | GCACCGGCGACAAATCAC | CCCAATTAAGATGACGGTCAAGA | 12 |  |
| *lp_1568* | transpeptidase (penicillin binding protein 2B) | TGTCGTACACCAAGGGTATTGG | CCGTCCGCTGCGTCAT | This study |  |
| *lp_1619* | serine/threonine protein kinase | GTCAGCGTGCCGCAACT | TCCCGACCGTGAGATGGT | This study |  |
| *lp_1669* | AraC family transcriptional regulator | GGACTTACAGCGGGCGTTTA | AACCCTGGTTGCAAAAATGC | 12 |  |
| *lp_1751* | transpeptidase-trans-glycosylase (penicillin binding protein 1A) | CGTTAAACTTGCGGTCTTTTCC | TCCTGCGCTTTGACTTTCAA | This study |  |
| *lp_2200* | transpeptidase, penicillin binding protein 2B | TGGATGGTCGGCAAGTGAA | TGAGCTTAACGCCCGTCAA | This study |  |
| *lp_2790* | 2-hydroxyacid dehydrogenase | GGTCAAATTGCCCACTTTCG | GCAAAGTTCGCCGCAAA | 14 |  |
| *lp_2940* | cell surface protein precursor, LPXTG-motif cell wall anchor | CCT GACCGGTTCGAGTGTTAG | CATCATGGCCCAGAAAATGAC | This study |  |
| *lp_2956* | tannase (tannin acylhidrolase) | CAACGGCGCCAATTCTG | GCCGGTCCTGGCAAATAAC | This study |  |
| *lp_2827* | Na(+)/H(+) antiporter | GGCCACATCGGTTTCGATT | CCATCGAGAGCGCCTAACT | 12 |  |
| *lp_3055* | copper transporting ATPase | ACGGCCCGTGGTATTCAA | TTACCCTCGATGGCTTGGAA | 14, 17 |  |
| *lp_3473* | alpha-L-rhamnosidase | TGGCGAGCTGTTTTTAGCAA | TCGCAGTGTCTACCGCACTT | 14, 17 |  |
| **Housekeeping and other internal control genes:** | |  |  |  |  |
| lp_2057 | D-lactate dehydrogenase | AACCGCGACAATGTTTTGATT | TTGTGAACGGCAGTTTCAGTGT | This study |  |
| lp_1963^c^ | DNA primase DnaG | TCCGGAAGCAGTCGTCAAG | TCGCCGGCAAGTCAATGT | This study |  |
| lp_0007 | DNA gyrase, A subunit | CCCGACAGCAACGTCTTCA | GGCAGCTGGCGTTTGTTT | This study |  |
| lp_1962 | RNA polymerase sigma factor RpoD | CGGATCCGCCAAATCG | CGTGATGGGTGGCGTAACTT | This study |  |
| lp_0006 | DNA gyrase, B subunit | CCCGGGTCGCTGCTAAG | TTTCCAAGCCACTCTTTTTTCG | This study |  |
| lp_2301 | recombinase A | CGGCGGGCAGAACAGAT | TTTCCAAGCCACTCTTTTTTCG | This study |  |
| lp_1021 | DNA-directed RNA polymerase subunit beta | GGGTGTGCCTTCTCGTATGAA | CAGCCATCCCCAAATGCA | This study |  |
| lp_rRNA01 | 16S ribosomal rRNA | GGGTAATCGGCCACATTGG | CTGCTGCCTCCCGTAGGA | This study |  |
